# The reverse congruency effect elicited by eye-gaze as a function of attention-deficit/hyperactivity disorder symptoms

**DOI:** 10.3389/fpsyg.2024.1377379

**Published:** 2024-06-14

**Authors:** Jeanette A. Chacón-Candia, Renato Ponce, Andrea Marotta

**Affiliations:** ^1^Department of Experimental Psychology, and Mind, Brain, and Behavior Research Center (CIMCYC), University of Granada, Granada, Spain; ^2^Department of Psychology, Sapienza University of Rome, Rome, Italy

**Keywords:** social attention, eye-gaze, ADHD, spatial Stroop, arrows

## Abstract

Individuals diagnosed with attention deficit/hyperactivity disorder (ADHD) have been found to have impairments in multiple aspects of social cognition, thus including the attentional processing of socially relevant stimuli such as eye-gaze. However, to date, it remains unclear whether only the social-specific but not the domain-general directional components, elicited by eye-gaze are affected by ADHD symptomatology. To address this issue, the present study aimed to investigate the impact of ADHD-like traits on the social-specific attentional processing of eye-gaze. To this purpose, we conducted an online experiment with a sample of 140 healthy undergraduate participants who completed two self-reported questionnaires designed to assess ADHD-like traits, and a social variant of an interference spatial task known to effectively isolate the social-specific component of eye-gaze. To make our research plan transparent, our hypotheses, together with the plans of analyses, were registered before data exploration. Results showed that while the social-specific component of eye-gaze was evident in the sample, no significant correlation was found between this component and the measured ADHD-like traits. These results appear to contradict the intuition that the attentional processing of the social-specific components of eye-gaze may be impaired by ADHD symptomatology. However, further research involving children and clinical populations is needed in order to clarify this matter.

## Introduction

Attention deficit hyperactivity disorder (ADHD) is a developmental condition characterized by significant levels of inattention, impulsivity and hyperactivity (Halperin et al., 1992). This condition has been found to have a detrimental impact on individuals’ academic, occupational, psychological and social spheres throughout their lifespan (Barkley et al., 1996; Wilens et al., 2002; Harpin, 2005).

Moreover, one of the most severe negative outcomes of ADHD is its impact on social functioning. Individuals with ADHD often struggle with social acceptance due to relatively lower social skills compared to their peers (Lee et al., 2012). Neurocognitive difficulties associated with ADHD, such as impulsivity and disinhibition, have been found to affect various aspects of social behavior, including Theory of Mind (ToM), emotional processing, prosocial behaviors, and empathy (for review, see Arango-Tobón et al., 2023).

In recent research, it has been observed that although the prevalence of an ADHD diagnosis from childhood persists into adulthood in approximately 43% of cases, the symptomatology associated with this disorder, particularly impulsivity and hyperactivity, tends to diminish (Di Lorenzo et al., 2021). Notably, a significant proportion of these symptoms transition into antisocial behaviors and substance use in adulthood. Additionally, while complete ADHD diagnoses in childhood reduce to about 15% in adulthood, a substantial 65% of these cases continue to exhibit symptoms that cause various impairments (Leffa et al., 2022). Of relevance, research on adults with ADHD has reported issues with friendships and poorer social interactions (Young et al., 2003; Kooij et al., 2010), as well as feelings of loneliness (Philipsen et al., 2009). These adults have also been found to experience less satisfying intimate relationships and marital adjustment (Eakin et al., 2004). Additionally, they report facing social isolation and a lifetime burden of the disorder, manifesting in consequences such as lower educational achievement. These cumulative problems across social, emotional, and occupational domains have been linked to a lower reported quality of life (Brod et al., 2012). In terms of social cognition, systematic reviews by Onandia-Hinchado et al. (2021) and Morellini et al. (2022) have also highlighted that domains such as empathy, emotion recognition, decision-making, and theory of mind are compromised in adults diagnosed with ADHD. This underscores the persistence of social cognition impairments, which are crucial aspects of our study’s focus.

In particular, individuals diagnosed with ADHD frequently encounter difficulties in recognizing socially relevant information conveyed through biologically relevant cues, such as eye-gaze. These challenges can lead to inappropriate responses within their social environment (Nejati, 2022). Understanding eye-gaze from early childhood is crucial for language acquisition, cultural learning, and the development of ToM processes, as eye-gaze conveys important information about individuals’ interests and mental states (Tomasello, 1995; Emery, 2000).

However, despite our understanding that individuals with ADHD often struggle to comprehend the messages transmitted through other people’s eyes, the implicit processing of this particular social cue (i.e., eye-gaze direction) within the population with ADHD symptomatology has only recently been studied. For example, Marotta et al. (2014) showed that individuals diagnosed with ADHD exhibit a selective impairment when orienting attention in response to eye-gaze direction. These authors found that individuals with ADHD reflexively orient toward locations previously signaled by stimuli with no biological relevance, such as arrows and peripheral cues. However, this population failed to automatically orient their attention in response to biologically relevant stimuli, such as eye-gaze.

These findings suggest that ADHD detaches the social-specific components associated with eye-gaze processing, while leaving the domain-general components of spatial cues/targets intact. Additionally, in a later study Marotta et al. (2017) also showed that individuals with ADHD were less sensitive to eye-gaze as a distracting stimulus in a Stroop-like task, compared to matched controls. However, no significant differences were observed between individuals with ADHD and controls when the distracting stimulus was an arrow (see also Polner et al., 2015). These findings may suggest that ADHD symptomatology may specifically impair the social-specific components associated with eye-gaze processing, while leaving the domain-general components of spatial cues/targets intact. However, this assumption must be clarified by using tasks that specifically focus on dissociating those components.

Hence, Marotta et al. (2018) proposed a variant of a spatial Stroop task that managed to capture the social-specific aspects of eye-gaze. These authors investigated the differences in the spatial interference effects elicited by socially relevant stimuli (i.e., eye-gaze) and non-socially relevant stimuli (i.e., arrow). They found that by manipulating the direction and location of eye-gaze and arrows, a dissociation in the effects elicited by these stimuli could be observed. When arrows were used as the target, a typical spatial Stroop effect (SCE) was observed, with participants responding faster to congruent trials (i.e., right-pointing arrows presented to the right) than to incongruent trials (i.e., right-pointing arrows presented to the left). However, when eye-gaze was the stimulus, the opposite effect was observed, with responses being faster for incongruent trials (i.e., right-gazing eyes presented to the left) than for congruent trials. These findings have been replicated across several studies (i.e., Edwards et al., 2020; Ishikawa et al., 2021; Román-Caballero et al., 2021a, 2021b; Narganes-Pineda et al., 2022), providing consistent evidence for this phenomenon known as the reversed congruency effect (RCE).

Marotta et al. (2018, 2019), have suggested that this RCE may be explained by the social significance of eye-gaze. On one hand, our sensibility to perceiving others’ faces or eyes is heightened when they make eye contact with us (i.e., Conty et al., 2006; Senju et al., 2008). In the Stroop task, when eye-gaze trials are incongruent, the eye-gaze stimulus is looking toward the center, establishing eye-contact with the participant, which leads to faster responses. Conversely, when eye-gaze trials are congruent, it appears that the eye-gaze stimulus is looking away from the participant, resulting in the opposite effect.

On the other hand, these authors suggest that the RCE could also be related to the “mentalizing” theory (Baron-Cohen et al., 1997), which refers to the ability to understand and interpretate another person’s intentions and mental states through their eyes, providing the possibility to anticipate their behavior. In particular, according to this perspective, participants do not misattribute the inward gaze as directed toward themselves, but rather perceive it as directed toward the fixation cross, to which they are also attending. This interpretation suggests that participants and the observed face engage in a shared episode of joint attention. In contrast, gaze discrimination is not facilitated when the eyes avert from the participant’s gaze direction, as joint attention is not established (see also, Edwards et al., 2020).

Thereby, following the intuition that the RCE found by Marotta et al. (2018) may indeed reflect the social-specific attentional processing of eye-gaze, and to investigate the processes underlying the attentional impairments to social stimuli in individuals with ADHD symptomatology, the aim of the present study was to investigate the effect of ADHD-like traits on the attentional system of the social-specific and domain-general processing of eye-gaze.

Growing body of evidence from various fields, including behavioral, neurocognitive, and genetic research, supports a dimensional perspective according to which ADHD is seen as an extreme manifestation of normal variation within the population, characterized by continuity in symptoms and underlying causes (Coghill and Sonuga-Barke, 2012; Sonuga-Barke et al., 2013). Impaired vigilance (Craig and Klein, 2019) and increased susceptibility to irrelevant distractions (Forster and Lavie, 2016) have shown positive correlations with ADHD symptoms in non-clinical samples. Additionally, executive functions have been found to correlate negatively with higher ADHD symptomatology in an adult non-clinical sample (Brown and Casey, 2016). Finally, and relevantly for this study, subclinical ADHD symptoms have also been associated with negative impacts on families, psychosocial problems, and lower life satisfaction (Gudjonsson et al., 2009; Cussen et al., 2012) and, in terms of social cognition, with face recognition impairment (Staff et al., 2021) and reduced emotional empathy (Groen et al., 2018).

Therefore, research on social attention impairment as function of ADHD symptom severity in community samples might shed light on processes of social cognition likely to be altered in ADHD (Coghill and Sonuga-Barke, 2012).

To this end, we first assessed ADHD-like traits by means of the Barkley Adult ADHD Rating Scale-IV (Barkley, 2011) and the Adult ADHD Self-Report Screening Scale for DSM-5 (Ustun et al., 2017). Second, we employed the spatial Stroop task developed by Marotta et al. (2018), which was designed to assess the attentional processing of the social-specific components of social stimuli, particularly eye-gaze. This study specifically explored the relationship between the RCE and ADHD-like traits in a substantial sample of healthy adults. Additionally, the SCE elicited by non-social stimuli (i.e., arrows) was also investigated.

We hypothesized that only the social-specific components elicited by eye-gaze, but not the domain-general component of the SCE, would be impaired by ADHD symptomatology. Specifically, we expected that a negative correlation would be observed only between ADHD-like traits and the RCE elicited by eye-gaze, but not between ADHD traits and the SCE elicited by arrows. The hypotheses for this experiment, together with the plans of analyses, were registered before data exploration in Open Science Framework.^1^

## Materials and method

### Participants

A total of 140 university students were recruited online to enroll in this study (Female = 130, Male = 8, Other = 2; mean age: 20.18 years). Prior to the experiment, all participants gave their informed consent to voluntarily participate in this research. All participants were naïve about the purpose of the study. The required sample size for this research has been estimated based on *a priori* power analysis, assuming an effect size of *r* = 0.31 (derived from Ishikawa et al., 2021) and a significance level of *α* = 0.05.

### Measures

#### Spatial interference task

The spatial interference task used in this study was created using the graphical experiment builder OpenSesame (Mathôt et al., 2012). The task followed a similar procedure to the one used by Marotta et al. (2018), although some modifications were made. Specifically, in this study, the central fixation cross was black, and the arrows and eyes stimuli were subtending in a fully white background. Unlike Marotta et al. (2018), in this study feedback was provided to participants only in the practice trials, and eyes and arrow targets were randomly presented within three experimental blocks of trials. Each trial began with the presentation of a central fixation cross for 1,000 ms. Subsequently, a pair of eyes or arrows signaling left or right appeared either on the left or right side of the fixation cross. Participants were instructed to discriminate as fast and accurately as possible the direction of the eyes or arrow stimuli by pressing the “M” key (with their right hand) when responding to targets signaling to the right and the “Z” key (with their left hand) when targets indicate the left, thus independently of the targets’ location. The targets remained on the screen until participants’ response or 2000 ms elapsed. Then, a blank screen was presented for 700 ms.

**Figure 1:**
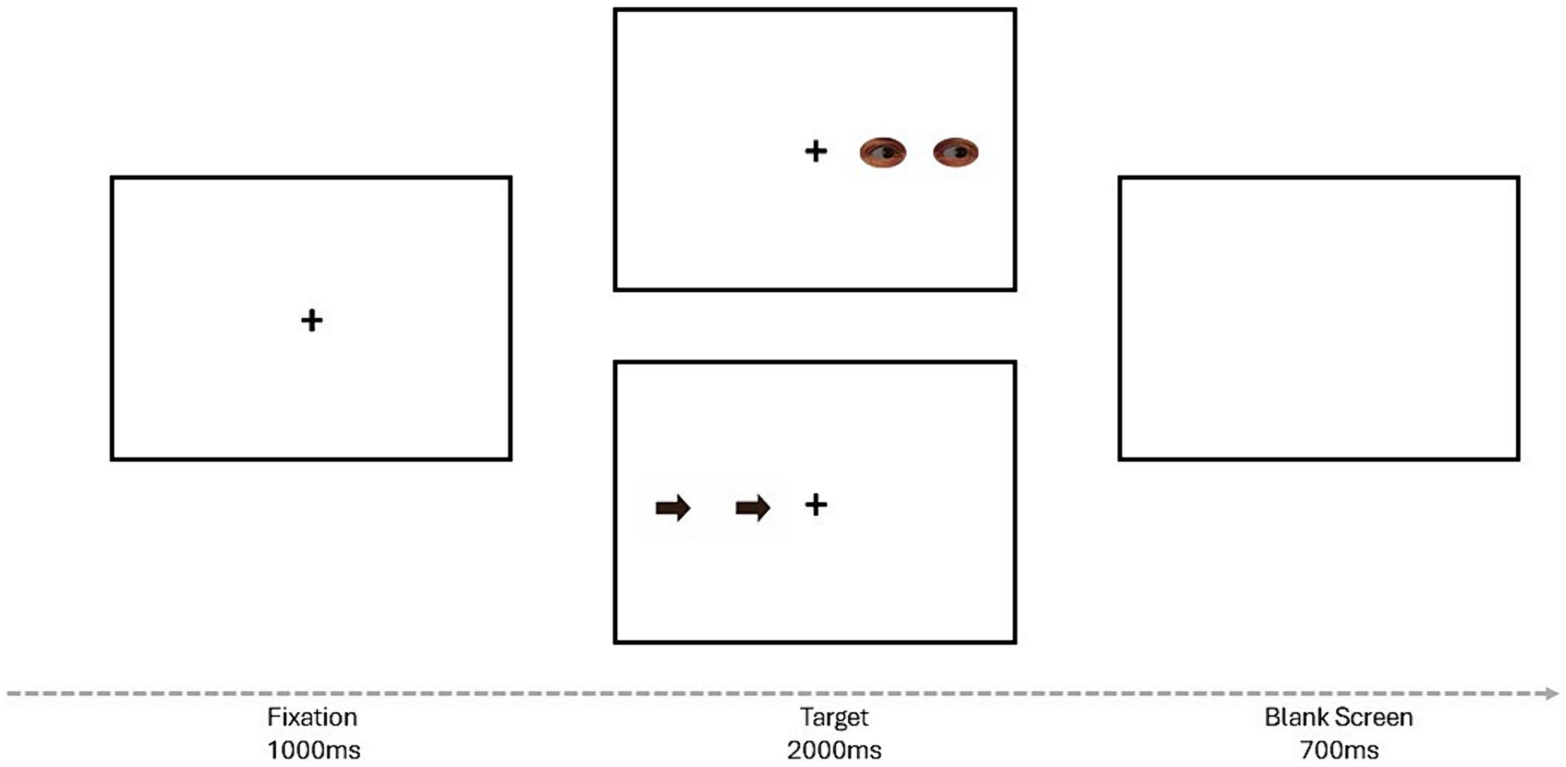
Schematic view of a trial sequence for both the arrow target and the gaze target conditions. The example represents: gaze target/congruent, and arrow target/incongruent conditions.

The targets were either congruent or incongruent. Congruent trials referred to target signaling in the same direction as the hemifield where they were presented (e.g., arrows pointing right presented on the right side of fixation). Incongruent trials involved targets signaling in the opposite direction of the hemifield where they were positioned (e.g., eyes gazing right presented on the left side of fixation). The task consisted of one practice block comprising 15 trials (in which participants received visual feedback for their performance), followed by three experimental blocks consisting of 48 trials each (where no feedback was given), summing up 159 trials in total. The target type, target direction, and target location were randomly interspersed within each block of trials (Figure 1).

#### Barkley Adult ADHD Rating Scale-IV

The self-report of the Barkley Adult ADHD Rating Scale-IV (BAARS-IV; Barkley, 2011) was designed to assess ADHD symptoms in adulthood. It includes two scales of nine items each to assess on the one hand individuals’ inattention and on the other individuals’ hyperactivity-impulsivity. Individuals must report how much they identify with several affirmations following a Likert scale ranging from 1 (never or rarely) to 4 (very often). In our sample, the scales demonstrated good internal consistency, with a reliability coefficients of *α* = 0.87 (*ω* = 0.87) for aBAARS-IV (inattention) and *α* = 0.76 (*ω* = 0.77) for aBAARS-IV (hyperactivity-impulsivity) and for the total scores of the two subscales, *α* = 0.88 (*ω* = 0.89), which is close to the *α* = 0.92 of the original BAARS-IV (Barkley, 2011). Barkley proposed the 95th percentile as a cut-off to identify individuals at high risk of ADHD.

#### Adult ADHD Self-Report Screening Scale for DSM-5

The Adult ADHD Self-Report Screening Scale for DSM-5 (ASRS-5; Ustun et al., 2017) was designed to assess the adult presentation of ADHD signs/symptoms according to the DSM-5 conceptualization (American Psychiatric Association, 2013). This scale is composed of six items, in which individuals are asked to respond to questions based on how they felt over the past 6 months (e.g., ‘how often do you put things off until the last minute?’) on a Likert scale ranging from 0 (never) to 4 (very often). The reliability of ASRS-5 in our sample was *α* = 0.66 (*ω* = 0.68), which falls within the range reported in the original study (Ustun et al., 2017), in which a cutoff score of 14 points was determined as the preferred threshold for screening purposes.

### General procedure

Participants completed the entire study online. They accessed the experiment through the university’s online platform for studies,^2^ where they were provided with a link hosting the experiment (thus, using the Lime Survey platform^3^). After expressing their consent to voluntarily participate in this study, individuals first completed the adult BAARS-IV (Barkley, 2011) and the ASRS-5 (Ustun et al., 2017) questionnaires. Upon completion of the questionnaires, participants were provided with a link that redirected them to the online version of the spatial interference task (hosted on a JATOS server). This study was conducted in conformity with the ethical standards of the Declaration of Helsinki and was approved by the Ethical Committee of the University of Granada (3232/CEIH/2023).

### Data analysis

For the spatial interference task, we conducted a 2(Target type) x 2(Congruency) repeated measures analysis of variance (ANOVA). Target type had two levels: gaze and arrow. Congruency had two levels: congruent and incongruent trials. Partial analyses of variance (ANOVAs) were conducted to analyse the interactions. Mean RTs and accuracy (as the mean percentage of errors) were considered separately as dependent variables. As in Marotta et al. (2018), RTs faster than 200 ms or slower than 1,300 ms, as well as incorrect responses, were excluded from the RT analysis.

To investigate the association between ADHD symptoms and spatial congruency effects, Pearson correlations were computed between the two questionnaires assessing ADHD symptoms (i.e., aBAARS-IV and ASRS-5) and both the SCE elicited by arrows and the RCE elicited by eye-gaze. For the SCE, the RTs for the “incongruent” trials were subtracted from the RTs for the “congruent” trials. For the RCE, the calculation was reversed.

To examine the relationship between ADHD symptoms and their potential influence on the spatial congruency effects, we also employed a linear mixed modeling (LMM) stepwise approach. We calculated the congruency effect by participant and target type and used it as a dependant variable. The lme4 package (Bates et al., 2015) in R was used for fitting the model, while the lmerTest package (Kuznetsova et al., 2017) facilitated backward stepwise model selection for both random and fixed effects. The initial saturated model included target type, as well as the normalized scores of the two BAARS subscales and the ASRS-5 as fixed effects. Random effects included intercepts for sex and participants. The stepwise model selection process employed both AIC and BIC criteria, complemented by likelihood ratio tests (LRT).

Additionally, to examine the cognitive performances of individuals with more pronounced ADHD-like traits versus those with fewer symptoms, we performed a separate analysis on participants at the highest and lowest extremes of the BAARS-IV and ASRS-5 total scores, specifically focusing on the first and last quartiles of our sample.

## Results

### Discrimination task

Mean RTs, standard deviations, and error percentages are presented in Table 1.

**Table 1:** Mean reaction times (RT), standard deviations (SD), and percentage of incorrect responses (%IR) as a function of target type and congruency.

	Arrow					Gaze			
Congruency	RT	*SD*	%IR	*SD*		RT	*SD*	%IR	*SD*
Congruent	483.25	78.59	2.01	3.76		577.05	90.5	5.93	7.04
Incongruent	506.68	79.335	4.65	6.05		566.31	87.89	7.47	6.92

### Reaction times

As in Marotta et al. (2018), RTs faster than 200 ms (0.03%) or slower than 1,300 ms (0.5%), as well as incorrect response trials (5%), were excluded from the RT analysis.

**Figure 2:**
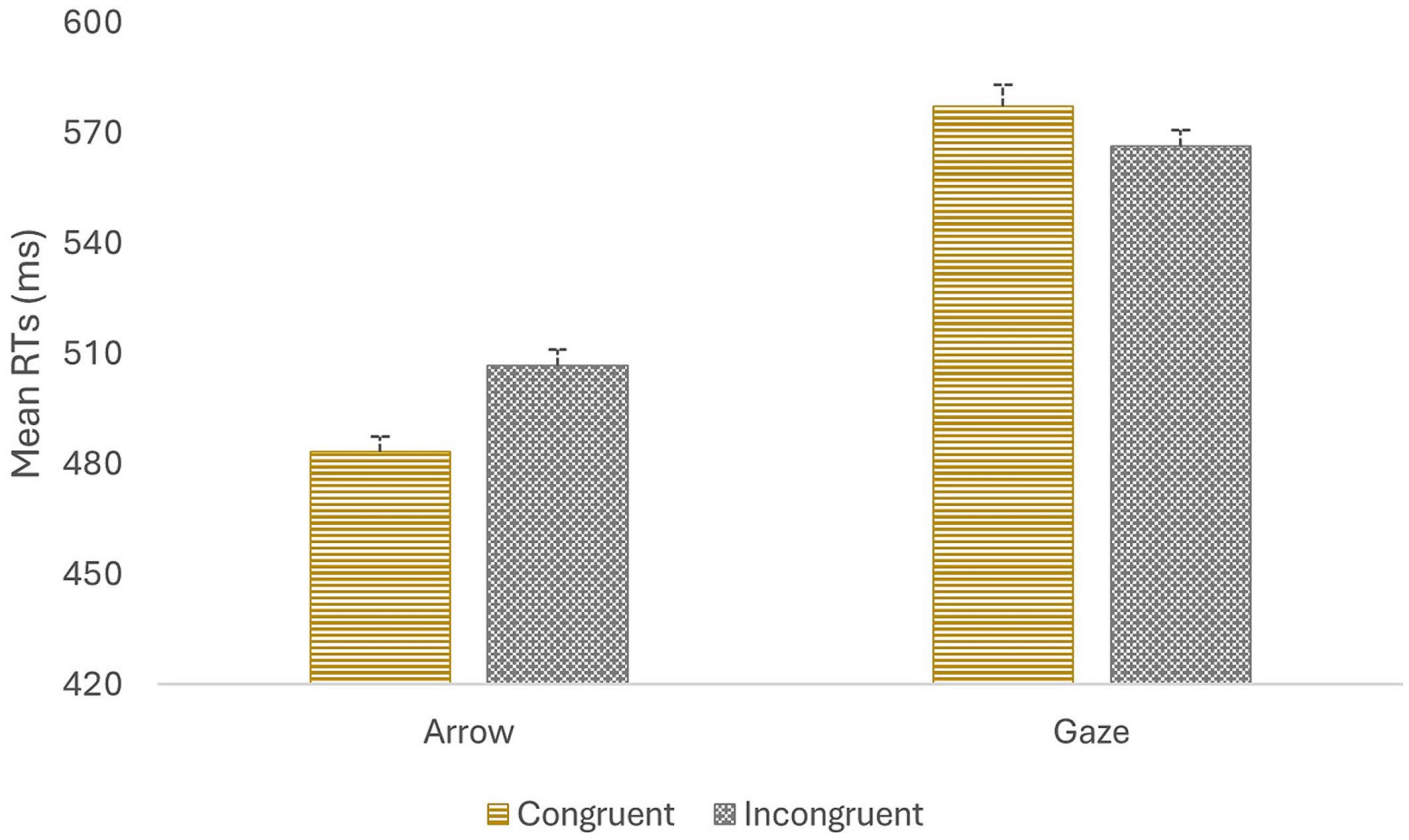
Mean reaction times for each target type and congruency conditions. Error bars represent the standard error of the mean, computed following Cousineau’s (2005) method to eliminate variability between participants.

The ANOVA revealed a main effect of target type, *F* (1, 139) = 556.65, *p*<0.001, *η*_p_^2^ = 0.80, indicating that participants responded faster to arrow targets than to gaze targets (495 vs. 572 ms). The main effect of congruency also reach significance, *F* (1, 139) = 5.170, *p* = 0.025, *η*_p_^2^ = 0.04, indicating that in general participants responded faster to congruent than to incongruent trials (530 vs. 536 ms). Importantly, the critical interaction of Target Type X Congruency was also significant, *F* (1, 139) = 67.08, *p*<0.001, *η*_p_^2^ = 0.33 (Figure 2). Partial ANOVA on each target type revealed that RTs were significantly faster on congruent trials (483 ms) than on incongruent trials (507 ms) when arrows were used as the targets*, F* (1, 139) = 77.55, *p*<0.001, *η*_p_^2^ = 0.36. In contrast, when gaze targets were used, RTs were significantly faster on incongruent trials (566 ms) than on congruent trials (577 ms), *F* (1, 139) = 6.71, *p* = 0.01, *η*_p_^2^ = 0.05.

### Errors

The ANOVA showed a significant main effect of target type *F* (1, 139) = 80.49, *p*<0.001, *η*_p_^2^ = 0.37, indicating that error rates were higher for gaze targets (6.7%), than for arrow targets (3.3%). The main effect of congruency was also significant *F* (1, 139) = 15.18, *p*<0.001, *η*_p_^2^ = 0.1, showing that participants made more errors on incongruent than on congruent trials (6.1% vs. 4%). The Target Type X Congruency interaction has not reached significance, *F* (1, 139) =2.29, *p* = 0.13, *η*_p_^2^ = 0.02.

### Correlation analysis

Pearson correlations were performed to test associations between ADHD symptoms (measured by the aBAARS-IV and ASRS-5 questionnaires), and the spatial congruency effects elicited by arrows (SCE) and eye-gaze (RCE). Overall, none of the relevant correlations reach significance. The results are presented in Table 2.

**Table 2:** Pearson correlations between ADHD symptoms questionnaires scores (aBAARS-IV and ASRS-5) and the spatial congruency effects elicited by arrows (SCE) and gaze (RCE).

		Arrow SCE	Gaze RCE
aBAARS-IV (total)	Pearson’s r	0.029	−0.039
	*p*-value	0.733	0.645
aBAARS-IV (inattention)	Pearson’s r	0.023	−0.040
	*p*-value	0.784	0.637
aBAARS-IV (hyperactivity-impulsivity)	Pearson’s r	0.029	−0.028
	*p*-value	0.732	0.739
ASRS-5	Pearson’s r	0.079	0.018
	*p*-value	0.355	0.836

### Linear mixed modeling (LMM) stepwise approach

Regarding the linear mixed model analysis, the evaluation of random effects through likelihood ratio tests (LRT) indicated that the random intercept for participant was essential; removing it significantly worsened the model fit, LRT = 11.95, df = 1, *p* < 0.001. However, the random intercept for sex did not significantly affect the model. For the fixed effects, the main effect of target type was significant, *F* (1, 139) = 67.08, *p* < 0.0001, *η*_p_^2^ = 0.33, justifying its retention in the model. Importantly, other fixed effects, including the scores of the BAARS subscales and the ASRS-5 were not significant (*p* > 0.05). Consequently, the resulting model, *Effect ~ target type + (1|id),* suggests that the questionnaires scores did not influence the outcomes regarding the spatial interference effect.

### Group comparison: extreme score analysis

For the BAARS-IV extreme scores, the analysis revealed a robust main effect of target type (*F* (1,68) = 346.14, *p* < 0.001, ηp^2 = 0.84), with participants responding more quickly to arrow targets compared to gaze targets (493 vs. 571 ms). The congruency effect was not significant, *F* > 1. However, a significant interaction between target type and congruency was observed (*F* (1, 68) = 36.09, *p* < 0.001, ηp^2 = 0.35), where response times (RTs) were faster in congruent trials (481 ms) than in incongruent trials (505 ms) for arrow targets. Conversely, for gaze targets, RTs were faster on incongruent trials (564 ms) compared to congruent trials (578 ms) (*F* (1, 68) = 5.83, *p* = 0.02, ηp^2 = 0.08). However, the interactions between high/low BAARS-IV scores and target type, high/low BAARS-IV scores and congruency, as well as the three-way interaction involving BAARS-IV scores, target type, and congruency, were all non-significant (all *F*s < 1).

For the ASRS-5 extreme scores, similarly, a significant main effect of target type was found (*F* (1,68) = 299.72, *p* < 0.001, ηp^2 = 0.82), with faster responses to arrow targets than gaze targets (499 vs. 572 ms). The main effect of congruency again did not reach significance, F > 1. A critical interaction of target type with congruency was significant (*F* (1, 68) = 30.34, *p* < 0.001, ηp^2 = 0.31). Partial ANOVAs for each target type revealed significantly faster RTs in congruent trials (488 ms) compared to incongruent trials (511 ms) for arrow targets (*F* (1, 68) = 34.07, *p* < 0.001, ηp^2 = 0.33). For gaze targets, RTs were faster in incongruent trials (566 ms) than in congruent trials (579 ms) (*F* (1, 68) = 4.49, *p* = 0.038, ηp^2 = 0.06). However, no significant interactions involving ASRS-5 scores were observed (all *F*s < 1).

## Discussion

In the present study, we explored the impact of ADHD-like traits on the attentional system for the processing of the social-specific components of eye-gaze. Participants first completed two self-report questionnaires specifically build to assessed ADHD symptomatology in adults [i.e., Barkley Adult ADHD Rating Scale-IV (Barkley, 2011); Adult ADHD Self-Report Screening Scale for DSM-5 (Ustun et al., 2017)]. Subsequently, participants performed a spatial Stroop task designed by Marotta et al. (2018) to assess the qualitatively different attentional mechanisms triggered by social and non-social stimuli (i.e., eye-gaze vs. arrow). This task has shown to effectively dissociate the social-specific component from the domain-general directional component of eye-gaze, thus when observing the RCE elicited by it.

Our results replicated the RCE observed by Marotta et al. (2018). When arrows were the targets, participants showed the classical interference effect, responding faster to congruent than to incongruent trials (i.e., arrow presented on the right side of the screen, pointing right). In contrast, when eye-gaze was the target, participants responded faster to incongruent than to congruent trials (i.e., eye-gaze presented on the right side of the screen, gazing to the left). However, both correlation analyses and the linear mixed modeling consistently indicated that ADHD-like traits, as measured by the BAARS-IV and ASRS-5, do not significantly influence the RCE, which contradict the intuition that the attentional processing of the social-specific components of eye-gaze may be impaired by ADHD symptomatology.

Importantly, our findings must be interpreted with caution given the limitations inherent in our study’s design. Firstly, our sample consisted of adult population, while most of the literature describing impairments in social cognition associated with ADHD symptomatology focuses on children and adolescent populations (see, Arango-Tobón et al., 2023).

In fact, Bora and Pantelis (2016) conducted a meta-analysis and found evidence that shows that social cognitive impairments in individuals diagnosed with ADHD tend to decrease from childhood to adulthood. These authors proposed that this improvement may be attributed to a catch-up in the neuronal development of higher-order functions, which are known to be delayed in individuals with ADHD when compared to typically-developed populations (Shaw et al., 2007; Sripada et al., 2014). Considering the implications of this meta-analysis, it is plausible to suggest that the ADHD-like traits observed in our adult participants may no longer significantly affect their social behavior. The developmental process, combined with the maturation of cognitive functions, could contribute to the amelioration of social cognitive impairments associated with ADHD. As the participants in our study were adults, it is reasonable to assume that their social cognition may have improved over time, aligning more closely with that of the typically developing population.

However, more recent studies have suggested that although a complete diagnosis of ADHD persists into adulthood in a minority of cases, a significant proportion of individuals continue to experience symptoms that cause impairments, with these symptoms fluctuating over the lifespan (Di Lorenzo et al., 2021; Leffa et al., 2022). Moreover, impairments in social cognition domains such as empathy, emotion recognition, decision making, and theory of mind remain prevalent in adults diagnosed with ADHD and have been documented to persist despite the general improvement in other symptoms (Onandia-Hinchado et al., 2021; Morellini et al., 2022). Additionally, subclinical variations of ADHD have been associated with significant psychosocial challenges, including negative family impacts and reduced life satisfaction (Gudjonsson et al., 2009; Cussen et al., 2012). This suggests that even minor ADHD-like traits in a non-clinical population can have profound implications, underscoring the relevance of studying these traits to understand their broader effects.

Moreover, while the current study provides important insights into the impact of ADHD-like traits on social-specific attentional processing, it does not include direct measures of social skills. Future research should aim to incorporate comprehensive assessments of social abilities to provide a deeper understanding of how individuals with ADHD-like traits manage social interactions and process social stimuli. Such investigations will help clarify whether the compensatory behaviors that adults develop to meet social norms or mask difficulties could significantly influence cognitive task performance.

Finally, given the nature of our study focusing on ADHD-like traits in a healthy adult population, rather than a clinical group, our findings offer an insight into how these traits might manifest differently from the typical developmental trajectory of ADHD.

The majority of the literature exploring social deficits in ADHD employs case–control designs, making comparisons between individuals diagnosed with ADHD and typically developing individuals. For instance, a recent study by Roselló et al. (2020) found that inattention, which is a core characteristic of ADHD, has a greater impact on functional impairments related to social behaviors in individuals diagnosed with ADHD compared to the typically developing individuals. Therefore, it is possible that the perception of being “inattentive” among our healthy participants may not necessarily be indicative of impairments in social cognition.

To gain a comprehensive understanding of the relationship between ADHD-like traits and social behavior, further studies are warranted. Future research could include longitudinal studies to track the developmental trajectory of social cognitive impairments in individuals with ADHD, as well as case–control studies to compare individuals diagnosed with ADHD to a control group. By examining these factors, we can obtain a more nuanced understanding of the impact of ADHD-like traits on social cognition across different developmental stages and populations.

## Data availability statement

The raw data supporting the conclusions of this article will be made available by the authors, without undue reservation.

## Ethics statement

The studies involving humans were approved by University of Granada 3232/CEIH/2023. The studies were conducted in accordance with the local legislation and institutional requirements. The participants provided their written informed consent to participate in this study.

## Author contributions

JC-C: Writing – review & editing. RP: Writing – review & editing. AM: Supervision, Writing – review & editing, Project administration.

## Footnotes

^1^ https://osf.io/8m9sk/

^2^ https://ugr-cimcyc.sona-systems.com

^3^ https://www.limesurvey.org/
